# COVID-19 Trends Among School-Aged Children — United States, March 1–September 19, 2020

**DOI:** 10.15585/mmwr.mm6939e2

**Published:** 2020-10-02

**Authors:** Rebecca T. Leeb, Sandy Price, Sarah Sliwa, Anne Kimball, Leigh Szucs, Elise Caruso, Shana Godfred-Cato, Matthew Lozier

**Affiliations:** ^1^CDC COVID-19 Response Team; ^2^Epidemic Intelligence Service, CDC.

Approximately 56 million school-aged children (aged 5–17 years) resumed education in the United States in fall 2020.* Analysis of demographic characteristics, underlying conditions, clinical outcomes, and trends in weekly coronavirus disease 2019 (COVID-19) incidence during March 1–September 19, 2020 among 277,285 laboratory-confirmed cases in school-aged children in the United States might inform decisions about in-person learning and the timing and scaling of community mitigation measures. During May–September 2020, average weekly incidence (cases per 100,000 children) among adolescents aged 12–17 years (37.4) was approximately twice that of children aged 5–11 years (19.0). In addition, among school-aged children, COVID-19 indicators peaked during July 2020: weekly percentage of positive SARS-CoV-2 test results increased from 10% on May 31 to 14% on July 5; SARS-CoV-2 test volume increased from 100,081 tests on May 31 to 322,227 on July 12, and COVID-19 incidence increased from 13.8 per 100,000 on May 31 to 37.9 on July 19. During July and August, test volume and incidence decreased then plateaued; incidence decreased further during early September and might be increasing. Percentage of positive test results decreased during August and plateaued during September. Underlying conditions were more common among school-aged children with severe outcomes related to COVID-19: among school-aged children who were hospitalized, admitted to an intensive care unit (ICU), or who died, 16%, 27%, and 28%, respectively, had at least one underlying medical condition. Schools and communities can implement multiple, concurrent mitigation strategies and tailor communications to promote mitigation strategies to prevent COVID-19 spread. These results can provide a baseline for monitoring trends and evaluating mitigation strategies.

School-aged children were stratified by age into two groups: children aged 5–11 years and adolescents aged 12–17 years. Confirmed COVID-19 cases were identified from individual-level case reports submitted by state health departments for the weeks beginning March 1–September 13, 2020.^†^ Confirmed cases had a positive real-time reverse transcription–polymerase chain reaction (RT-PCR) test result for SARS-CoV-2, the virus that causes COVID-19. COVID-19 case data for all children were analyzed to examine demographic characteristics, underlying conditions,^§^ hospitalization, ICU admission, and death. Trends were analyzed using CDC report date^¶^ to calculate a daily 7-day moving average, aggregated by week. Analyses are descriptive; statistical comparisons were not performed.

To examine trends in laboratory testing volume and percentage of positive test results, data from COVID-19 electronic laboratory data were used. SARS-CoV-2 RT-PCR test results were obtained for the weeks beginning May 31–September 13, 2020 from COVID-19 electronic laboratory reporting data submitted by state health departments (37 states); when age was unavailable in state-submitted data, information from data submitted directly by public health, commercial, and reference laboratories (13 states, Puerto Rico, and the District of Columbia) were used.** Data represent test results, not number of persons tested; specimen collection date or test order date was used for analysis.^††^ The weekly percentage of positive SARS-CoV-2 RT-PCR test results was calculated nationally for each U.S. Department of Health and Human Services (HHS) Region^§§^ as the number of positive test results divided by the sum of positive and negative test results.

During March 1–September 19, 2020, a total of 277,285 laboratory-confirmed cases of COVID-19 in school-aged children were reported in the United States, including 101,503 (37%) in children aged 5–11 years and 175,782 (63%) in adolescents aged 12–17 years (Table). Overall, 50.8% were in females (aged 5–11 years = 49.4%; aged 12–17 = 51.6%). Among 161,387 (58%) school-aged children with COVID-19 and complete information on race/ethnicity, 42% were Hispanic/Latino (Hispanic), 32% were non-Hispanic White (White), and 17% were non-Hispanic Black (Black). Hispanic children accounted for 46% of cases among younger children and 39% among adolescents; White children accounted for 26% of cases in younger children and 36% in adolescents.^¶¶^ Weekly incidence among school-aged children increased from March 1, peaking at 37.9 cases per 100,000 the week of July 19 (aged 5–11 years = 25.7; aged 12–17 years = 51.9), plateaued at an average of 34 per 100,000 during July 26–August 23, decreased to 22.6 per 100,000 the week of September 6, and rebounded to 26.3 per 100,000 the last week for which data are available (Figure 1) (Supplementary Figure 1, https://stacks.cdc.gov/view/cdc/94150). Trends in incidence were similar among both age groups. Incidence among adolescents was approximately double that among younger children throughout the reporting period. During May–September, average weekly incidence among adolescents was 37.4 cases per 100,000 compared with 19.0 per 100,000 for younger children.

**TABLE:** Demographic characteristics and underlying conditions among school-aged children aged 5–11 years and 12–17 years* with positive test results for SARS-CoV-2 (N = 233,474) — United States, March 1–September 19, 2020

Characteristic	Age group, no. (%)		
	All (N = 277,285)	5–11 yrs (n = 101,503)	12–17 yrs (n = 175,782)
**Sex^†^**			
Female	140,755 (50.8)	50,096 (49.4)	90,659 (51.6)
Male	136,530 (49.2)	51,407 (50.6)	85,123 (48.4)
Median age, yrs	13	8	15
**Symptom status**			
Yes	161,751 (58.3)	56,917 (56.1)	104,834 (59.6)
No	12,806 (4.6)	5,985 (5.9)	6,821 (3.9)
Missing/Unknown	102,728 (37.0)	38,601 (38.0)	64,127 (36.5)
**Race/Ethnicity** ^§^			
Hispanic/Latino	67,275 (41.7)	27,539 (45.9)	39,736 (39.2)
White, non-Hispanic	52,229 (32.4)	15,503 (25.8)	36,726 (36.2)
Black, non-Hispanic	27,963 (17.3)	11,315 (18.8)	16,648 (16.4)
A/PI, non-Hispanic	4,541 (2.8)	1,932 (3.2)	2,609 (2.6)
AI/AN, non-Hispanic	3,044 (1.9)	1,342 (2.2)	1,702 (1.7)
Multiracial/Other race	6,335 (3.9)	2,421 (4.0)	3,914 (3.9)
Unknown**^¶^**	115,898 (N/A)	41,451 (N/A)	74,447 (N/A)
**Underlying condition**			
Any	7,738 (2.8)	2,396(2.4)	5,342 (3.0)
Chronic lung disease**	4,214 (54.5)	1,441 (60.1)	2,773 (51.9)
Disability^††^	714 (9.2)	251 (10.5)	463 (8.7)
Immunosuppression	526 (6.8)	193 (8.1)	333 (6.2)
Diabetes mellitus	476 (6.2)	88 (3.7)	388 (7.3)
Psychological/Psychiatric	445 (5.8)	60 (2.5)	385 (7.2)
Cardiovascular disease	363 (4.7)	128(5.3)	235 (4.4)
Current/Former smoker^§§^	334 (4.3)	11 (0.5)	323 (6.0)
Severe obesity (BMI ≥40 kg/m^2^)	315 (4.1)	70 (2.9)	245 (4.6)
Chronic kidney disease	116 (1.5)	47 (2.0)	69 (1.3)
Hypertension	94 (1.2)	13 (0.5)	81 (1.5)
Autoimmune	87 (1.1)	16 (0.7)	71 (1.3)
Chronic liver disease	64 (0.8)	14 (0.6)	50 (0.9)
Substance abuse/use	34 (0.4)	0 (0.0)	34 (0.6)
Other^¶¶^	1,326 (17.1)	419 (17.5)	907 (17.0)
**Outcome**			
Hospitalized***	3,240 (1.2)	1,021 (1.0)	2,219 (1.3)
ICU admission^†††^	404 (0.1)	145 (0.1)	259 (0.1)
Died^§§§^	51 (<0.1)	20 (<0.1)	31 (<0.1)

**Abbreviations:** A/PI = Asian/Pacific Islander; AI/AN = American Indian/Alaska Native; BMI = body mass index; COVID-19 = coronavirus disease 2019; ICU = intensive care unit.; N/A = not available.

* Age was missing for 1.9% of all persons with positive test results; the proportion aged 5–17 years cannot be determined.

^†^ Among 281,116 persons aged 5–17 years with COVID-19, sex was missing, unknown, or other for 3,831 (1.4%).

^§^ Persons for whom ethnicity was missing (i.e., not reported as either “Hispanic” or “non-Hispanic”) were categorized has having missing race/ethnicity.

^¶^ Missing data were excluded from the denominator for calculating percentage of each racial/ethnic group. Missing rates did not differ by age group. Multiracial/other race includes persons reported as American Indian/Alaskan Native, Native Hawaiian or other Pacific Islander, multiracial, and persons of another race without further specification.

** Chronic lung disease includes asthma, emphysema, and chronic obstructive pulmonary disease (COPD).

^††^ Disability includes neurologic and neurodevelopmental disorders (e.g., seizure disorders, autism spectrum disorders, and developmental delay), intellectual and physical disabilities, vision or hearing impairment, genetic disorders and inherited metabolic disorders, and blood disorders (e.g., sickle cell disease and hemophilia).

^§§^ Checked the box on the case report form for either “current smoker” or “former smoker.”

^¶¶^ Other includes conditions not listed elsewhere, conditions with no specific autoimmune etiology, endocrine disorders other than diabetes (e.g., polycystic ovarian disease, hypothyroidism, and hyperthyroidism), gastrointestinal disorders (e.g., gastritis or gastroesophageal reflux), obstructive sleep apnea, allergies/atopy, anemia (etiology not specified), history of cancer in remission, and other conditions that did not fall under the specified categories.

*** Hospitalization status. 5–11 years: missing/unknown = 44,300 (43.6%); 12–17 years: missing/unknown = 79,411 (45.2%).

^†††^ ICU admission status. 5–11 years: missing/unknown = 90,405 (89.0%); 12–17 years: missing/unknown = 154,662 (88.0%).

^§§§^ Mortality status. 5–11 years: missing/unknown = 47,006 (46.3%); 12–17 years: missing/unknown = 83,479 (47.5%).

**FIGURE 1:**
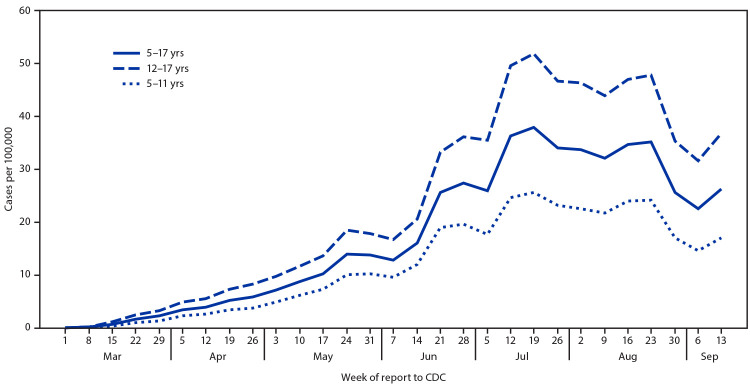
COVID-19 incidence* among school-aged children aged 5–11 years (N = 101,503) and 12–17 years (N = 175,782), by week — United States, March 1–September 19, 2020^†^ **Sources:** CDC COVID-19 case report form. https://wwwn.cdc.gov/nndss/covid-19-response.html. CDC National Notifiable Disease Surveillance System. https://wwwn.cdc.gov/nndss. **Abbreviation:** COVID-19 = coronavirus disease 2019. * Incidence = cases per 100,000, calculated using 2018 population from https://datacenter.kidscount.org/. ^†^ Data included through September 19, 2020, so that each week has a full 7 days of data.

Weekly SARS-CoV-2 laboratory test volume among school-aged children more than tripled, from 100,081 tests performed during the week beginning May 31 to a peak of 322,227 during the week beginning July 12, then decreased to approximately 260,000 during August and rebounded in September; test volume was higher among adolescents than younger children (Figure 2) (Supplementary Figure 1, https://stacks.cdc.gov/view/cdc/94150) (Supplementary Figure 2, https://stacks.cdc.gov/view/cdc/94151). The percentage of positive SARS-CoV-2 laboratory test results increased for both age groups from May 31 and peaked during the week beginning July 5; percentage of positive test results then decreased among both age groups. Since August 23, the percentage of positive SARS-CoV-2 laboratory test results plateaued at 7% among adolescents and continued to decrease among younger children.

**FIGURE 2:**
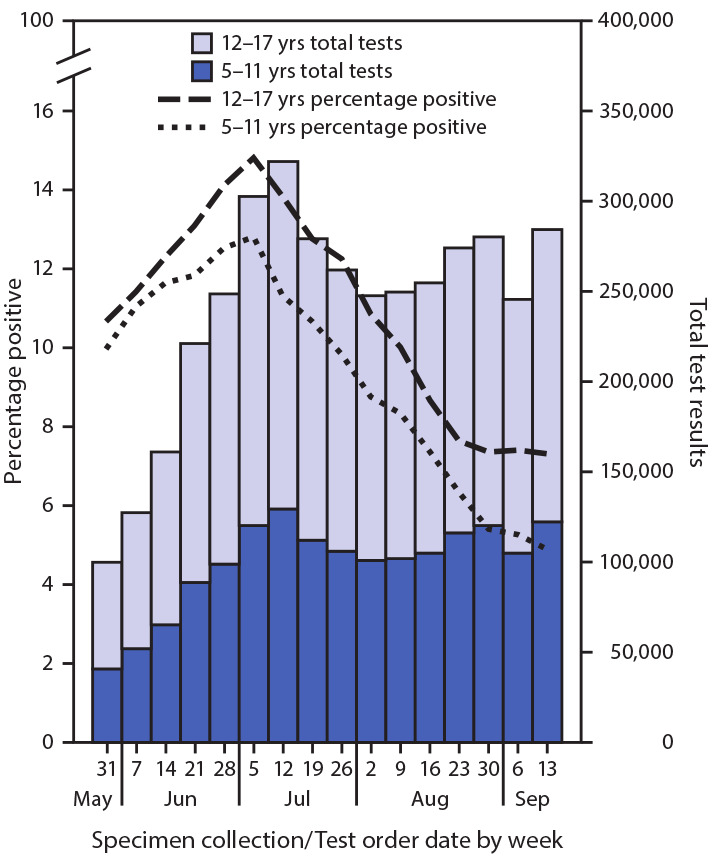
Percentage of SARS-CoV-2 reverse transcription–polymerase chain reaction (RT-PCR) tests with positive results and test volume, by week for school-aged children aged 5–11 years and 12–17 years — United States, May 31–September 19, 2020* **Abbreviation:** COVID-19 = coronavirus disease 2019. * From COVID-19 electronic laboratory reporting data submitted by state health departments for 37 states and from data submitted directly by public health, commercial, and reference laboratories for 13 states, Puerto Rico, and the District of Columbia, using specimen collection or test order date. The data represent percentage of tests, not of individual persons, with a positive result and include RT-PCR tests but not antigen or point-of-care tests.

HHS Regions 6, 4, and 9 had the highest weekly percentage of positive test results, peaking during the week of July 5 at 24% (Region 6), 18% (Region 4), and 17% (Region 9), and all declined to approximately 8% the week beginning September 13 (Supplementary Figure 2, https://stacks.cdc.gov/view/cdc/94151). In Region 1, weekly percentage of positive tests decreased from 8% during the week beginning May 31 to <2% during the week beginning September 13. In Region 9, the percentage of positive test results was similar over time in both age groups; in Regions 5 and 7, although the percentage of positive test results were initially similar in both age groups, beginning in early June (Region 7) and mid-June (Region 5), the percentage of positive test results in adolescents exceeded that among younger children.

Among school-aged children with laboratory-confirmed COVID-19, 58% reported at least one symptom, 5% reported no symptoms, and information on symptoms was missing or unknown for 37% (Table). Overall, 3,240 (1.2%) school-aged children with COVID-19 were hospitalized, including 404 (0.1%) who required ICU admission. Fifty-one (<0.01%) school-aged children died of COVID-19. Among school-aged children with complete information on race/ethnicity who were hospitalized (2,473 [76%]) or admitted to an ICU (321 [80%]), Hispanic ethnicity was most commonly reported (45% and 43%, respectively), followed by Black (24% and 28%, respectively) and White (22% and 17%, respectively) races.

Among school-aged children with COVID-19, at least one underlying condition was reported for 7,738 (3%), including approximately 3% of adolescents and 2% of younger children. Among those with an underlying condition, chronic lung disease, including asthma, was most commonly reported (55%), followed by disability*** (9%), immunosuppressive conditions (7%), diabetes (6%), psychological conditions (6%), cardiovascular disease (5%), and severe obesity (4%). At least one underlying condition was reported for 16% of school-aged children who were hospitalized for COVID-19, 27% of those admitted to an ICU, and 28% of those who died.

## Discussion

As education resumes and some schools begin in-person learning for the 2020–21 academic year, it is critical to have a baseline for monitoring trends in COVID-19 infection among school-aged children. Since March, a period during which most U.S. schools conducted classes virtually or were closed for the summer, the incidence among adolescents was approximately double that in younger children. Although mortality and hospitalization in school-aged children was low, Hispanic ethnicity, Black race, and underlying conditions were more commonly reported among children who were hospitalized or admitted to an ICU, providing additional evidence that some children might be at increased risk for severe illness associated with COVID-19 (*1*–*4*).^†††^ Acute COVID-19 and multisystem inflammatory syndrome in children (MIS-C) have been reported to disproportionately affect Hispanic and Black children (*3*,*4*). Implementing multiple, concurrent mitigation strategies and tailored communications about the importance of promoting and reinforcing behaviors that reduce spread of COVID-19 (e.g., wearing masks, maintaining a social distance of ≥6 feet, and frequent handwashing) can reduce COVID-19 spread in schools and communities.

Monitoring trends in multiple indicators of COVID-19 could inform mitigation measures to prevent COVID-19 spread.^§§§^ COVID-19 incidence increased from March to July, and SARS-CoV-2 test volume and weekly percentage of positive test results among school-aged children increased from late May to July. During March through May, widespread shelter-in-place orders were in effect, and most U.S. schools transitioned to online learning. In June and July, when community mitigation measures were relaxed in some areas, incidence increased more rapidly. Recent evidence that monthly COVID-19 incidence increased approximately threefold among persons aged 0–19 years since May and was highest among young adults aged 20–29 years during July, suggests that young persons might be playing an increasingly important role in community transmission (*5*,*6*). The percentage of positive test results in school-aged children also varied within and across HHS regions. Variations in percentage of positive tests might indicate differences in community transmission rates. School studies suggest that in-person learning can be safe in communities with low SARS-CoV-2 transmission rates^¶¶¶^ (*7*) but might increase transmission risk in communities where transmission is already high.****

The findings in this report are subject to at least four limitations. First, these data might underestimate the actual incidence of disease among school-aged children, because testing was frequently prioritized for persons with symptoms, and asymptomatic infection in children is common (*8*). These data are also from a single reporting system and therefore might not represent the total number of cases and deaths in school-aged children reported in the United States (*1*). Second, findings on race/ethnicity, symptom status, underlying conditions, and outcomes should be interpreted with caution; these data had high rates of missing or unknown values. Third, because of delays in reporting, trend data might lag behind actual disease transmission dates. Because of missing symptom onset and specimen collection dates, COVID-19 cases are presented by the date each case was reported to CDC, and surveillance artifacts can exist as a result of batch reporting by states.^††††^ Finally, laboratory data presented here underrepresent the volume of laboratory tests reported in some states, because state reporting of laboratory data and case surveillance is not uniform.^§§§§^

These findings can provide a baseline for monitoring national trends. Monitoring at the local-level could inform decision-makers about which mitigation strategies are most effective in preventing the spread of COVID-19 in schools and communities (*6*,*9*). CDC’s considerations for schools outline important mitigation strategies for safer reopening for in-person learning.^¶¶¶¶^ Schools and communities should implement multiple concurrent preventive strategies and adjust mitigation depending on local levels of transmission to reduce COVID-19 disease risk for students, teachers, school staff members, families and the community.

**Summary**

**What is already known about this topic?**

Children aged <10 years can transmit SARS-CoV-2 in school settings, but less is known about COVID-19 incidence, characteristics, and health outcomes among school-aged children (aged 5–17 years) with COVID-19.

**What is added by this report?**

Since March, 277,285 COVID-19 cases in children have been reported. COVID-19 incidence among adolescents aged 12–17 years was approximately twice that in children aged 5–11 years. Underlying conditions were more common among school-aged children with severe outcomes related to COVID-19. Weekly incidence, SARS-CoV-2 test volume, and percentage of tests positive among school-aged children varied over time and by region of the United States.

**What are the implications for public health practice?**

It is important for schools and communities to monitor multiple indicators of COVID-19 among school-aged children and layer prevention strategies to reduce COVID-19 disease risk for students, teachers, school staff, and families. These results can provide a baseline for monitoring trends and evaluating mitigation strategies.

## Footnotes

* https://nces.ed.gov/fastfacts/display.asp?id=372.

† During the COVID-19 pandemic, CDC receives both aggregate and individual (i.e., line-list) counts of cases and deaths from reporting jurisdictions. CDC official counts of cases and deaths, released daily at https://www.cdc.gov/coronavirus/2019-ncov/cases-updates/cases-in-us.html, are aggregate counts from reporting jurisdictions. Some jurisdictions electronically submit standardized information for individual cases of COVID-19 to CDC using the Human Infection with 2019 Novel Coronavirus Case Report Form (COVID-19 Case Report Form) developed for the CDC COVID-19 response (https://www.cdc.gov/coronavirus/2019-ncov/php/reporting-pui.html) or the CDC National Notifiable Diseases Surveillance System (NNDSS) (https://wwwn.cdc.gov/nndss/covid-19-response.html). Individual-level case report data were available for approximately 69% of the aggregate number of confirmed cases. Cases reported without sex or age data and in persons repatriated to the United States from Wuhan, China, or the Diamond Princess cruise ship were excluded from this analysis.

§ Underlying conditions were defined based on the categories included in the COVID-19 Case Report Form including diabetes mellitus, hypertension, severe obesity (body mass index [BMI] ≥40 kg/m^2^), cardiovascular disease, chronic renal disease, chronic liver disease, chronic lung disease (asthma, emphysema, and chronic obstructive pulmonary disease [COPD]), other (specified) chronic diseases, other (specified) underlying condition or risk behavior, immunosuppressive conditions, autoimmune conditions, being a current or former smoker, substance abuse or misuse, disability (neurologic, neurodevelopmental, intellectual, physical, vision or hearing impairment, and psychological/psychiatric condition). Although obesity in children is not generally defined using BMI, these data are drawn from the NNDSS case report form in which severe obesity is defined as noted.

¶ CDC report date is the date the case was reported to CDC by the state health department. If CDC report date was missing, report date was populated with the earliest date in a series of variables submitted by the jurisdiction, including hospital or ICU admission and discharge date, diagnosis date, symptom onset and resolution dates, and positive specimen dates. As of August 9, 2020, approximately 10% of reported COVID-19 confirmed cases in the 50 states and District of Columbia had no available date information; it cannot be estimated when these were reported to CDC during May–August 2020 (the analytic period for this study).

** COVID-19 Electronic Laboratory Reporting data submitted by state health departments from all laboratories performing SARS-CoV-2 RT-PCR testing were used for 37 states (Alabama, Alaska, Arizona, Arkansas, Colorado, Connecticut, Florida, Georgia, Hawaii, Idaho, Illinois, Indiana, Iowa, Kansas, Kentucky, Louisiana, Maryland, Massachusetts, Michigan, Minnesota, Montana, Nebraska, Nevada, New Hampshire, New Jersey, North Carolina, Oregon, Pennsylvania, South Carolina, South Dakota, Tennessee, Texas, Utah, Vermont, Virginia, West Virginia, and Wisconsin). SARS-CoV-2 RT-PCR testing data from a subset of public health, commercial, and reference laboratories were used for the District of Columbia and 14 states/territories (California, Delaware, Maine, Mississippi, Missouri, New Mexico, New York, North Dakota, Ohio, Oklahoma, Puerto Rico, Rhode Island, Washington, and Wyoming). The data from the public health, commercial, and reference laboratories represent approximately 50% of all tests. The data might not include results from all testing sites within a jurisdiction (e.g., point-of-care test sites) and therefore reflect the majority of, but not all, SARS-CoV-2 RT-PCR tests in the United States. The data represent laboratory test totals, not individual persons tested, and exclude antibody and antigen tests.

†† For COVID-19 electronic laboratory reporting data, state was assigned using the state health department reporting the test (available for 100% of tests), and specimen collection date was used to assign date (available for approximately 98% of tests); those with missing specimen collection date were excluded. Within data submitted directly by public health, commercial, and reference laboratories, state was assigned using patient location for 96% of tests; provider location was substituted for 1%, and records with both location fields missing (3%) were excluded; order date was used for 80% of tests, specimen collection date was substituted for 19%, and records with both date fields missing (1%) were excluded.

§§ The HHS Office of Intergovernmental and External Affairs hosts 10 regional offices that directly serve state and local organizations. https://www.hhs.gov/about/agencies/iea/regional-offices/index.html.

¶¶ In 2018, children of Hispanic/Latino ethnicity comprised 26% of children aged 5–11 years and 24% of adolescents aged 12–17 years; children of non-Hispanic Black race comprised 14% of children aged 5–11 years and 14% of adolescents 12–17 years; and children of non-Hispanic White race comprised 50% of children aged 5–11 years and 52% of adolescents aged 12–17 years in the United States. https://datacenter.kidscount.org/data/customreports/1/8446.

*** Disability included neurologic or neurodevelopmental disorders, intellectual or physical disability, and vision or hearing impairment.

††† https://www.cdc.gov/coronavirus/2019-ncov/need-extra-precautions/other-at-risk-populations.html.

§§§ https://www.cdc.gov/coronavirus/2019-ncov/community/schools-childcare/indicators.html.

¶¶¶
https://www.cdc.gov/coronavirus/2019-ncov/community/community-mitigation.html .

**** https://arxiv.org/pdf/2006.14158.pdf.

†††† https://www.cdc.gov/coronavirus/2019-ncov/covid-data/faq-surveillance.html.

§§§§ Percentage positive for laboratory data for some states relied on data reported directly to CDC from public health laboratories and a sample of six large commercial laboratories.

¶¶¶¶ Four cross-cutting strategies to reduce the spread of COVID-19 are outlined in CDC’s Community Mitigation Framework: promote behaviors that prevent spread, maintain healthy environments, maintain healthy operations, and prepare for when someone gets ill. https://www.cdc.gov/coronavirus/2019-ncov/community/community-mitigation.html.
