# *Erratum*: Vol. 69, No. 39

**DOI:** 10.15585/mmwr.mm695152a5

**Published:** 2021-01-01

In the report, “COVID-19 Trends Among School-Aged Children — United States, March 1–September 19, 2020,” the percentage of school-aged children with underlying conditions was calculated using school-aged children for whom one or more underlying conditions was reported as the denominator. Information on underlying conditions is not reported for the vast majority (approximately 80%) of school-aged children included in CDC’s National Notifiable Diseases Surveillance System, so using the total population as the denominator to calculate the percent of children with underlying conditions would substantially underestimate the prevalence of underlying conditions. To correct this, the percentage of school-aged children with underlying conditions was recalculated using school-aged children for whom underlying condition status was known as the denominator (i.e., a “yes” or “no” on case report form, excluding “unknown” and no information).

On page 1410, in the first paragraph, the seventh sentence should have read “Underlying conditions were more common among school-aged children with severe outcomes related to COVID-19: among school-aged children who were hospitalized, admitted to an intensive care unit (ICU), or who died, **23%**, **38%**, and **33%**, respectively, had at least one **underlying condition**.

On page 1412, in the second column, the first paragraph should have read “Among school-aged children with COVID-19, **data about** underlying **conditions were** reported for **59,851 (22%). At least one underlying condition was reported for 17,319 (29%) of those with known underlying condition status,** including **11,333** adolescents and **5,986** younger children. Among those with r**eported data about** underlying **conditions**, chronic lung disease, including asthma, was most commonly reported (**7%**), followed by disability**^†††^** (**1%**), immunosuppressive conditions (**0.9%**), diabetes (**0.8%**), psychological conditions (**0.7%**), cardiovascular disease (**0.6%**), and severe obesity (**0.5%**). At least one underlying condition was reported for **23%** of school-aged children who were hospitalized for COVID-19, **38%** of those admitted to an ICU, and **33%** of those who died.”

On page 1412, there were multiple errors in the Table for the “Underlying conditions” section. Corrections to Table footnotes are bolded. The corrected Table is as follows:

**TABLE:** Demographic characteristics and underlying conditions among school-aged children aged 5–11 years and 12–17 years* with positive test results for SARS-CoV-2 (N = 233,474) — United States, March 1–September 19, 2020

Characteristic	Age group, no. (%)		
	All (N = 277,285)	5–11 yrs (n = 101,503)	12–17 yrs (n = 175,782)
**Sex^†^**			
Female	140,755 (50.8)	50,096 (49.4)	90,659 (51.6)
Male	136,530 (49.2)	51,407 (50.6)	85,123 (48.4)
Median age, yrs	13	8	15
**Symptom status**			
Yes	161,751 (58.3)	56,917 (56.1)	104,834 (59.6)
No	12,806 (4.6)	5,985 (5.9)	6,821 (3.9)
Missing/Unknown	102,728 (37.0)	38,601 (38.0)	64,127 (36.5)
**Race/Ethnicity^§^**			
Hispanic/Latino	67,275 (41.7)	27,539 (45.9)	39,736 (39.2)
White, non-Hispanic	52,229 (32.4)	15,503 (25.8)	36,726 (36.2)
Black, non-Hispanic	27,963 (17.3)	11,315 (18.8)	16,648 (16.4)
A/PI, non-Hispanic	4,541 (2.8)	1,932 (3.2)	2,609 (2.6)
AI/AN, non-Hispanic	3,044 (1.9)	1,342 (2.2)	1,702 (1.7)
Multiracial/Other race	6,335 (3.9)	2,421 (4.0)	3,914 (3.9)
Unknown^¶^	115,898 (N/A)	41,451 (N/A)	74,447 (N/A)
**Underlying conditions**			
Known underlying condition status**	59,851 (21.6)	21,505 (21.2)	38,346 (21.8)
Any underlying condition	17,319 (28.9)	5,986 (27.8)	11,333 (29.6)
Chronic lung disease**^††^**	4,214 (7.0)	1,441 (6.7)	2,773 (7.2)
Disability**^§§^**	714 (1.2)	251 (1.2)	463 (1.2)
Immunosuppression	526 (0.9)	193 (0.9)	333 (0.9)
Diabetes mellitus	476 (0.8)	88 (0.4)	388 (1.0)
Psychological/psychiatric	445 (0.7)	60 (0.3)	385 (1.0)
Cardiovascular disease	363 (0.6)	128 (0.6)	235 (0.6)
Current/Former smoker**^¶¶^**	334 (0.6)	11 (0.1)	323 (0.8)
Severe obesity (BMI ≥40)	315 (0.5)	70 (0.3)	245 (0.6)
Chronic kidney disease	116 (0.2)	47 (0.2)	69 (0.2)
Hypertension	94 (0.2)	13 (0.1)	81 (0.2)
Autoimmune	87 (0.1)	16 (0.1)	71 (0.2)
Chronic liver disease	64 (0.1)	14 (0.1)	50 (0.1)
Substance abuse/use	34 (0.1)	0 (0.0)	34 (0.1)
Other***	10,907 (18.2)	4,009 (18.6)	6,898 (18.0)
**Outcome**			
Hospitalized**^†††^**	3,240 (1.2)	1,021 (1.0)	2,219 (1.3)
ICU admission**^§§§^**	404 (0.1)	145 (0.1)	259 (0.1)
Died**^¶¶¶^**	51 (<0.1)	20 (<0.1)	31 (<0.1)

**Abbreviations**: A/PI = Asian/Pacific Islander; AI/AN = American Indian/Alaska Native; BMI = body mass index; COVID-19 = coronavirus disease 2019; N/A = not available.

* Age was missing for **1.4%** of all persons with positive test results; the proportion aged 5–17 years cannot be determined.

^†^ Among **242,259** persons aged 5–17 years with COVID-19, sex was missing, unknown, or other for **8,785 (3.6%)**.

^§^ Persons for whom ethnicity was missing (i.e., not reported as either “Hispanic” or “non-Hispanic”) were categorized has having missing race/ethnicity.

^¶^ Missing data were excluded from the denominator for calculating percentage of each racial/ethnic group. Missing rates did not differ by age group. Multiracial/other race includes persons reported as American Indian/Alaskan Native, Native Hawaiian or other Pacific Islander, multiracial, and persons of another race without further specification.

** Status of underlying conditions known for 59,851 school-aged children: 21,505 aged 5–11 years and 38,346 aged 12–17 years. Status was classified as “known” if any of the following conditions were reported as present or absent: diabetes mellitus, hypertension, severe obesity (BMI ≥40 kg/m^2^), cardiovascular disease, chronic kidney disease, chronic liver disease, chronic lung disease, immunocompromising condition, autoimmune condition, disability, psychological/psychiatric condition, current or former smoker, substance abuse or use, and other underlying medical condition not otherwise specified. Those with known underlying condition status were used as the denominator for the remaining underlying conditions in the table.

**^††^** Chronic lung disease includes asthma, emphysema, and chronic obstructive pulmonary disease (COPD).

**^§§^** Disability includes neurologic and neurodevelopmental disorders (e.g., seizure disorders, autism spectrum disorders, and developmental delay), intellectual and physical disabilities, vision or hearing impairment, genetic disorders and inherited metabolic disorders, and blood disorders (e.g., sickle cell disease and hemophilia).

**^¶¶^** Checked the box on the case report form for either “current smoker” or “former smoker.”

*** Other includes conditions not listed elsewhere, conditions with no specific autoimmune etiology, endocrine disorders other than diabetes (e.g., polycystic ovarian disease, hypothyroidism, and hyperthyroidism), gastrointestinal disorders (e.g., gastritis or gastresophageal reflux), obstructive sleep apnea, allergies/atopy, anemia (etiology not specified), history of cancer in remission, and other conditions that did not fall under the specified categories.

**^†††^** Hospitalization status. 5–11 years: missing/unknown = 44,300 (43.6%); 12–17 years: missing/unknown = 79,411 (45.2%).

**^§§§^** ICU admission status. 5–11 years: missing/unknown = 90,405 (89.0%); 12–17 years: missing/unknown = 154,662 (88.0%).

**^¶¶¶^** Mortality status. 5–11 years: missing/unknown = 47,006 (46.3%); 12–17 years: missing/unknown = 83,479 (47.5%).

